# Administration with Quinoa Protein Reduces the Blood Pressure in Spontaneously Hypertensive Rats and Modifies the Fecal Microbiota

**DOI:** 10.3390/nu13072446

**Published:** 2021-07-17

**Authors:** Huimin Guo, Yuqiong Hao, Xin Fan, Aurore Richel, Nadia Everaert, Xiushi Yang, Guixing Ren

**Affiliations:** 1 Institute of Crop Sciences, Chinese Academy of Agricultural Sciences, Beijing 100081, China; guohuimin0208@sina.com (H.G.); haoyuqiong334@163.com (Y.H.); fx_926@163.com (X.F.); renguixing@caas.cn (G.R.); 2 Gembloux Agro-Bio Tech, University of Liege, 5030 Gembloux, Belgium; a.richel@ulg.ac.be (A.R.); nadia.everaert@uliege.be (N.E.)

**Keywords:** antihypertension, gastrointestinal digestion, ACE inhibitory peptides, gut dysbiosis, microbiota modification

## Abstract

Despite the well-established role of quinoa protein as the source of antihypertensive peptides through in vitro enzymolysis, there is little evidence supporting the in vivo antihypertensive effect of intact quinoa protein. In this study, in vivo study on spontaneously hypertensive rats (SHRs) was conducted by administering quinoa protein for five weeks. Gastrointestinal content identification indicated that many promising precursors of bioactive peptides were released from quinoa protein under gastrointestinal processing. Quinoa protein administration on SHRs resulted in a significant decrease in blood pressure, a significant increase in alpha diversity, and microbial structure alternation towards that in non-hypertension rats. Furthermore, blood pressure was highly negatively correlated with the elevated abundance of genera in quinoa protein-treated SHRs, such as *Turicibacter* and *Allobaculum*. Interestingly, the fecal microbiota in quinoa protein-treated SHRs shared more features in the composition of genera with non-hypertension rats than that of the captopril-treated group. These results indicate that quinoa protein may serve as a potential candidate to lower blood pressure and ameliorate hypertension-related gut dysbiosis.

## 1. Introduction

Hypertension is a major public health concern around the world, and it has been linked to an increased risk of cardiovascular events and end-stage renal disease [1]. In addition to drug treatment, dietary intervention is also a well-established strategy to lower blood pressure [1,2]. As a macro nutrition in the human diet, protein plays an important role in human health, and it has been proposed to associate with blood pressure in hypertensive patients and animal models [3,4]. Ait-Yahia et al. [5] reported that consumption of the fish protein diet could result in an attenuation of hypertension development in spontaneously hypertensive rats (SHRs). A study in humans showed that systolic blood pressure (SBP) and diastolic blood pressure (DBP) were significantly lowered by the intake of whey protein after 12 weeks [6]. Furthermore, many research and clinical trials have provided extensive scientific evidence for the application of milk derived peptides for the prevention and treatment of hypertension [7,8]. The potential factors responsible for the antihypertensive effect of protein are reported to be multifaceted, including the amino acid composition, bioactive peptides, interactions with the gut microbiota, and so on [3,9].

Bioactive peptides can be released from dietary protein as a result of in vivo gastrointestinal digestion. There is an increasing number of researches focus on the antihypertension effect of food protein-derived peptides [10]. Angiotensin-converting enzyme (ACE) plays a significant role in blood pressure regulation via the renin-angiotensin and kinin-kallikrein systems, making it one of the promising physiological targets for antihypertensive treatment [11]. Various dietary protein has been employed for the generation of ACE inhibitory peptides, including animal products, marine organisms, and plants [12].

Gut microbiota is a community of microorganisms in the gastrointestinal tract, and they are closely related to the health of the host. In recent years, considerable information has been accumulated on the relationship between the gut microbiota and hypertension, which demonstrated a significant reduction in microbial richness and diversity both in hypertensive animals and humans, and suggested the potential role of microbiota modulation in the treatment of hypertension [13,14]. Some studies have reported interactions between gut microbiota and antihypertensive treatments, including drugs and dietary intervention [15,16,17]. Gut microbiota can make a significant response to dietary intervention, and long-term diets play a decisive role in the microbial community structure [18], which is expected to be an alternative method in the treatment of hypertension.

Quinoa (*Chenopodium quinoa* Willd.), a pseudocereal that originated in South America, has been recognized as an excellent source of dietary protein due to its high level of protein and well-balanced amino acids composition. Several quinoa protein hydrolysates that prepared by Alcalase, pepsin, trypsin, bromelain, etc., have been reported to possess in vitro ACE inhibitory activity [19,20,21,22,23], and some of them have demonstrated excellent antihypertensive activity in SHRs [22,23]. However, the in vivo antihypertensive effect of intact quinoa protein remains unclear. The main aim of the work was to strengthen knowledge on the potential contribution of quinoa protein on the regulation of blood pressure. LC–ESI–Q–TOF–MS was used to analyze the peptides produced by in vivo digestion of quinoa protein. Additionally, we assessed the alternation of the fecal microbiota in response to antihypertensive treatments in SHRs.

## 2. Materials and Methods

### 2.1. Preparation of Quinoa Protein

Protein ingredients were extracted from *Mengli-I* quinoa seeds by a previously reported method [24]. In brief, defatted quinoa flour was suspended in distilled water (1:10, *w/w*), protein solubilization was carried out at pH 9.0 by stirring for 60 min, and precipitation was carried out at pH 4.5 followed by centrifugation at 10,000× *g* for 30 min. The precipitates were resuspended in water, neutralized, and lyophilized. As reported in our previous study, the yield of quinoa protein extracts and protein purity were 6.51% and 85.23%, respectively [24].

### 2.2. Animals and Experimental Design

The experiments were performed following the recommendations of the Guide for the Care and Use of Laboratory Animals of the National Institutes of Health and were approved by the Experimental Animal Ethics Committee of Pony Testing International Group. Eight-week-old male SHRs and Wistar Kyoto rats (WKYs) (220–250 g body weight (BW)) were purchased from Beijing Vital River Laboratory Animal Technology Co., Ltd. (Beijing, China). All animals were acclimatized for two weeks in the animal facility (one rat per cage) under specific pathogen-free conditions, with free access to water and sterilized food (AIN-93M Diet, Beijing Huafukang Bioscience Co., Ltd., Beijing, China).

In the short-term study, SHRs were randomly divided into five groups (5 rats per group). These groups received a saline solution, 10 mg/kg BW captopril, 100 mg/kg, 200 mg/kg, and 400 mg/kg BW quinoa protein, respectively, through oral administration, using syringes [24]. Each dose of quinoa protein and captopril was dissolved in 1 mL of saline solution.

In the long-term study, SHRs were assigned into three groups (7 rats per group) of SHR–S, SHR–Q, and SHR–C. These groups were orally administered once daily for five weeks by saline, 200 mg/kg BW quinoa protein, and 10 mg/kg BW captopril, respectively. Seven WKYs were assigned as a control group by administration of saline.

### 2.3. Measurement of Blood Pressure 

The rats were trained to adapt to the measurement of blood pressure by the tail-cuff method during the acclimatization period. Rats were kept in a warm environment (37 °C) for 15 min, and then transferred to a standard equipment with heating pad and restrainer. SBP and DBP were measured using the tail-cuff method with a pulse transducer [25]. In the short-term study, the SBP and DBP were measured at 0, 2, 4, 6, and 8 h after oral administration. In the long-term study, the blood pressure was weekly measured at 4 h after oral administration, based on the results of short-term study. Each value of blood pressure was an average reading of five measurements.

### 2.4. Collection of Fecal Samples

After the long-term oral administration, the rats (SHR–S, SHR–Q, SHR–C, and WKY–S) were induced to defecate by abdominal massage. Fresh fecal samples were collected in sterile tubes and stored at −80 °C. To assess the baseline profile of fecal microbiota, fecal samples were also collected from SHRs (seven randomly chosen rats, SHR–B) and WKYs (WKY–B) before experimental intervention.

### 2.5. Collection of Digested Samples 

The rats were fasted overnight before receiving oral administration of quinoa protein extract (dissolved in 1 mL of sterile saline solution) at a dose of 200 mg/kg BW. Then, the rats were euthanized at 0.5, 1, and 2 h after oral administration. Their stomach contents, small intestine (including duodenum, jejunum, and ileum) contents, and colon contents were collected at each time and dissolved in 1 mL cold saline solution. After centrifugation at 4000× *g* for 20 min, the supernatants were frozen in liquid nitrogen and stored before peptides identification.

### 2.6. LC–ESI–Q–TOF–MS Analysis 

After filtration through a 0.45 mum filter (Merck Millipore, Darmstadt, Germany), digested samples were loaded onto a C18 trap column (5 μm, 5 × 0.3 mm, Agilent Technologies, Santa Clara, CA, USA) and then separated by a C18 analytical column (75 μm × 150 mm, 3 μm particle size, 100 Å pore size, Eksigent). The peptides identification was carried out by TripleTOF 5600 mass spectrometer (AB SCIEX, Framingham, MA, USA) as described in the previous study [24]. The MS properties were set as follows: scan range, 350-1500 m/z; accumulation time, 0.25 s; ion source gas 1, 25 psi; curtain gas, 25 psi; ion spray voltage floating, 2700 V; interface heater temperature, 150 °C; collision energy, 10 V; and declustering potential, 100 V. The data processing was done using Proteome Discoverer 2.2 (Thermo Fisher Scientific, Sunnyvale, CA, USA) and Mascot 2.3 (Matrix Science, Boston, MA, USA).

### 2.7. Gut Microbiota Analysis

Microbial DNA in fecal samples was extracted using the E.Z.N.A.^®^ soil DNA Kit (Omega Bio-Tek, Norcross, GA, USA) according to the manufacturer’s recommendations. The DNA was quantified by a NanoDrop 2000 spectrophotometer (Thermo Fisher Scientific, Wilmington, NC, USA). Purified DNA was used to amplify the V3-V4 region of 16S rRNA genes by thermocycler PCR system (GeneAmp 9700, ABI, Vernon, CA, USA). The resulted PCR products were extracted from a 2% agarose gel and further purified using the AxyPrep DNA Gel Extraction Kit (Axygen Biosciences, Union City, CA, USA) and quantified using QuantiFluor™-ST (Promega, Madison, WI, USA), according to the manufacturer’s protocol. Purified amplicons were pooled in equimolar and paired-end sequenced (2 × 300) on an Illumina MiSeq platform (Illumina, San Diego, CA, USA). The raw FASTQ files were processed using QIIME 2. 

### 2.8. Statistical Analysis

Blood pressure values were expressed as the mean ± standard error of the mean (SEM) and analyzed using the one-way analysis of variance (ANOVA) with Tukey test. Beta diversity was measured via Bray–Curtis and UniFrac distances and then visualized by nonmetric multidimensional scaling (NMDS) using the R phyloseq package. Analysis of similarity (ANOSIM) was performed to evaluate the significant differences in observed clustering using the R Vegan package, and pairwise comparison was statistically assessed using one-way permutation multivariate analysis of variance (PERMANOVA) with 999 permutations. Comparisons of alpha diversity and species relative abundance were made using ANOVA and Tukey post hoc test in case of normal distribution, or Kruskal–Wallis with Dunn’s multiple comparison test in case of abnormal distribution. The linear discriminant analysis (LDA) effect size (LEfSe) method was used to select the different species between groups with the threshold of 2.0. Correlations between the fecal microbiota and blood pressure were carried out using Spearman’s rank correlation coefficient and visualized by the R pheatmap package. 

## 3. Results and Discussion

### 3.1. Identification of Peptides in Digested Samples

In vivo digestion is a spatiotemporal and dynamic process that involves food degradation, nutrient absorption, and transport processes. The dietary protein is digested into peptides by pepsin in the stomach and further metabolized in the small intestine into smaller peptides and free amino acids, which can be transported and progressively absorbed along the small intestine [26]. Enzymes from gut microbiota in the small and large intestine also play a role in dietary protein metabolism [27,28]. In this study, gastrointestinal contents following ingestion of quinoa protein in rats were described by performing LC–ESI–Q–TOF–MS analysis and matching with the quinoa protein in the Mascot database. The list of all the peptides identified in digested samples was summarized in Supplementary Table S1. A total of 395 spectra were detected and identified in the gastrointestinal contents, including 164 different peptides originating from quinoa protein. Specifically, 108, 53, and 17 peptides were identified from the gastric contents collected at 0.5, 1, and 2 h after protein ingestion, respectively. Only a few or no peptides were identified in the small intestine and colon digesta of rats. It was speculated that intestinal digestion and absorption lowered the abundance of existing peptides in the gastric contents, resulting in the peptides poorly detectable with the experimental procedure in this study. On the other hand, it could also be partly explained by the undetectability of new smaller peptides produced by further enzymatic hydrolysis in the small intestine. Peptide composed of less than six amino acids is a limitation in protein identification by MS analysis because it is difficult to map the peptide to the precursor protein [27]. Although no peptide from the quinoa protein was detected in colon contents, we could not be sure that no quinoa protein was delivered to the colon. Indeed, even highly digested protein or peptides may partly escape digestion and absorption in the small intestine and then are transferred to the large intestine, where the residual peptides can be metabolized by intestinal microbiota [29].

The identified peptides were mapped on their parent protein sequences; 13S globulin seed storage protein 2-like, legumin A-like, and 11S globulin seed storage protein 2-like were the main protein precursors of identified peptides (Table 1), and the peptides that originating from these protein sequences contributed for 32.15%, 21.01%, and 20.25% of the total peptides, respectively. These three proteins also ranked in the top three on protein sequence coverage, with 32.47%, 41.34%, and 44.25%, respectively. Within the protein sequences, partial regions were highly recovered by the identified peptides, and several fragments were frequently detected such as YRNAIMAPHYN and TRGDIIAIPPGAVH (Supplementary Table S1). As for the poorly recovered regions in matched protein sequences, they may be extensively hydrolyzed into small peptides, or these fragments are resistant to gastrointestinal digestion and are too large to be detected by MS methodology [30].

We took matched sequences of main quinoa protein as precursors; profiles of potential biological activity were conducted by BIOPEP online predictive analysis. In silico analysis showed that quinoa protein contained a large number of bioactive peptides that described as ACE inhibitors in the BIOPEP database (Table 1), and most of these ACE inhibitory peptides were dipeptides or tripeptides, which can be easily absorbed in the intestine. In addition, FHPFPR and NIFRPF, previously reported ACE inhibitory peptides from quinoa protein [24], appeared as subfragments of SLSLPNFHPFPR and INNIFRPFA in the present study, indicating that quinoa protein fragments are good precursors of ACE inhibitory peptides. 

### 3.2. Antihypertensive Effect of Quinoa Protein on SHRs

**Figure 1:**
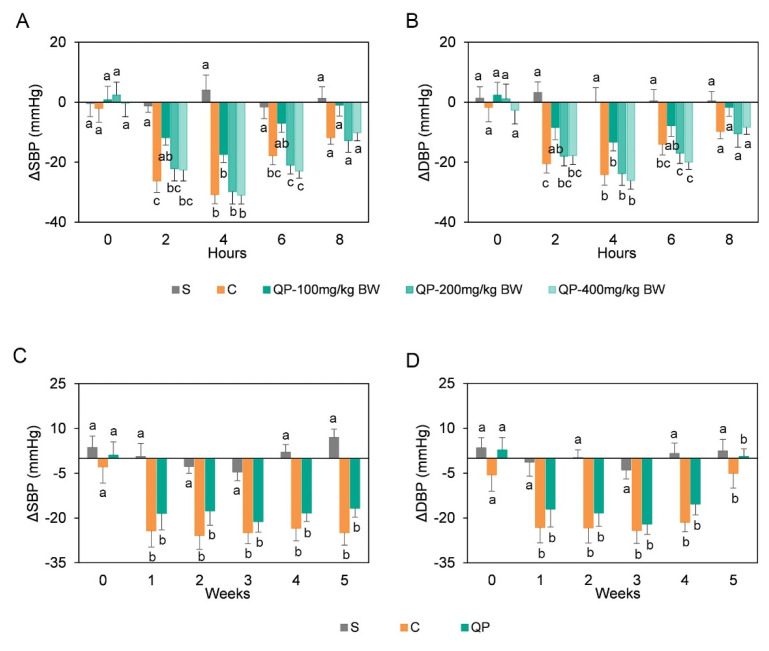
Oral administration of quinoa protein has an impact on the blood pressure of spontaneously hypertensive rats (SHRs). In the short-term study, systolic blood pressure (SBP) (**A**) and diastolic blood pressure (DBP) (**B**) in SHRs were measured at 0, 2, 4, 6, and 8 h after oral administration of saline solution (S), captopril (C), 100 mg/kg BW, 200 mg/kg BW, and 400 mg/kg BW quinoa protein (QP). In the long-term study, SBP (**C**) and DBP (**D**) of SHRs were measured during five-week administration of saline solution (S), captopril (C), and 200 mg/kg BW quinoa protein (QP). Based on the initial blood pressure value before intervention, change values of blood pressure (ΔSBP and ΔDBP) were expressed as the mean ± standard error of the mean. At each time point, columns with different letters are significantly different at *p* < 0.05.

The treatment of hypertension during the early stage is important to attenuate the development of hypertension and prevent future complications of hypertension [31]. Moreover, lifestyle changes, including dietary factors, were recommended as initial management strategies in hypertension [32]. Therefore, SHRs at 10 weeks-old were used in this experiment to investigate the effect of quinoa protein on early-stage hypertension. Before the experimental intervention, the initial SBP and DBP of all the SHRs were observed to be not significantly different from each other (*p* > 0.05). In the short-term study (Figure 1A,B), blood pressure of SHRs significantly decreased (*p* < 0.05) at 2 h after oral administration of captopril and descended to its lowest level at 4 h. Blood pressure of SHRs significantly decreased at 4 h after administration of 100 mg/kg BW quinoa protein, and there was no significant difference at 2 and 6 h compared to the untreated group. Quinoa protein at the doses of 200 mg/kg BW and 400 mg/kg BW showed great potential in lowering blood pressure of SHRs between 2 and 6 h after oral administration, and led to the largest decrease of blood pressure at 4 h. Compared to the captopril treatment group, there was no significant difference (*p* > 0.05) in blood pressure for SHRs treated with quinoa protein at the dose of 200 or 400 mg/kg BW. Accordingly, a long-term administration of quinoa protein was performed and the blood pressure conditions were shown in Figure 1C,D. Both administrations of quinoa protein and captopril resulted in a significant decrease (*p* < 0.05) in SBP and DBP of the SHRs in the long-term study. However, no significant difference (*p* > 0.05) of blood pressure was found between the quinoa protein and captopril-treated rats. 

The food intake and body weight of rats were not significantly affected by quinoa protein administration, and the blood pressure of normotensive WKYs was not significantly affected by quinoa protein administration at 400 mg/kg BW (data not shown). Much research has focused on the antihypertension characteristics of protein from the quinoa and its enzymolysis products. For example, RGQVIYVL, a peptide from quinoa bran albumin, showed a significant antihypertensive effect in SHRs at a concentration of 100 or 150 mg/kg BW [22]. As reported in our previous study [23], quinoa protein hydrolysate produced by simulated gastrointestinal digestion showed a potent ACE inhibitory activity, and induced a significant decrease in blood pressure of SHRs between 2 and 10 h after oral administration at the dose of 200 or 400 mg/kg BW. It could be found that both intact and hydrolyzed quinoa protein at the dose of 200 or 400 mg/kg BW have led to a similar maximal blood pressure reduction with 10 mg/kg BW captopril. The present study demonstrated that quinoa protein under in vivo gastrointestinal digestion also exerted a great impact on blood pressure in SHRs. It is presumed that some bioactive peptides may be produced during the digestion of protein and participate in the regulation of blood pressure in the SHRs.

### 3.3. Microbial Taxonomic Compositions of SHRs and WKYs

**Figure 2:**
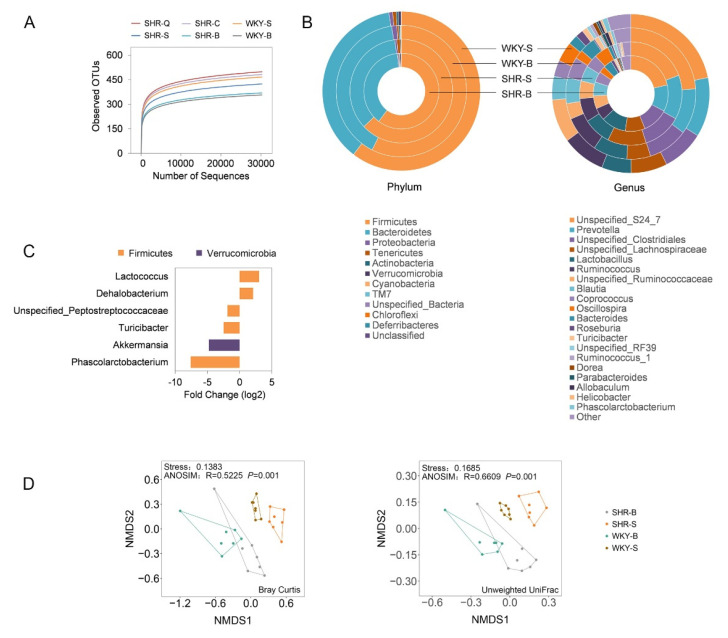
Significant differences in fecal microbial compositions between spontaneously hypertensive rats (SHRs) and Wistar Kyoto rats (WKYs). SHR–B and WKY–B: rats at baseline (10-week-old); SHR–S and WKY–S: rats administrated by saline solution for five weeks (15-week-old); SHR–Q and SHR–C: rats orally administrated by quinoa protein and captopril for five weeks (15-week-old), respectively. (**A**) Rarefaction curves. (**B**) Relative abundance of fecal microbiotas at the phylum and genus levels in rats. Four layers of donuts from the center to the outside represent the relative abundance of fecal microbiotas in SHR–B, SHR–S, WKY–B, and WKY–S, respectively. (**C**) Differentially abundant genera by DESeq2 (P_adj_ < 0.01) when SHR–S vs WKY–S. (**D**) β-diversity of the fecal microbiota based on nonmetric multidimensional scaling (NMDS) analysis.

In total, 42 fecal samples from rats were subjected to 16S rRNA gene sequencing. An average of 42,272 sequences (ranging from 30,192 to 58,249) per biological sample was detected after filtered, denoised, merged, and chimera deleted. These sequences were assigned to an average of 443 OTUs per sample. Rarefaction curves presented in Figure 2A indicated sequencing depth was adequate to capture a representative microbial diversity. 

Before (10-week-old) and after (15-week-old) long-term intervention, fecal samples from SHR–S and WKY–S were collected, and microbial compositions of these groups of samples were presented in Figure 2B at the taxonomic levels of phylum and genus. The main phyla in all samples were Firmicutes and Bacteroidetes, together accounting for above 97% of all classifiable sequences in rats. The longitudinal comparison revealed that the microbial composition showed an age-dependent change in both SHRs and WKYs. To identify the genus with different abundance between SHRs and WKYs at 15-week-old, the DESeq analysis was performed and those genera with an adjusted *p*-value of less than 0.01 were presented in Figure 2C. SHRs were characterized by a higher abundance of Lactococcus and Dehalobacterium, while had poor relative abundances of Phascolarctobacterium, Akkermansia, Turicibacter, and Unspecified_Peptostreptococcaceae.

**Figure 3:**
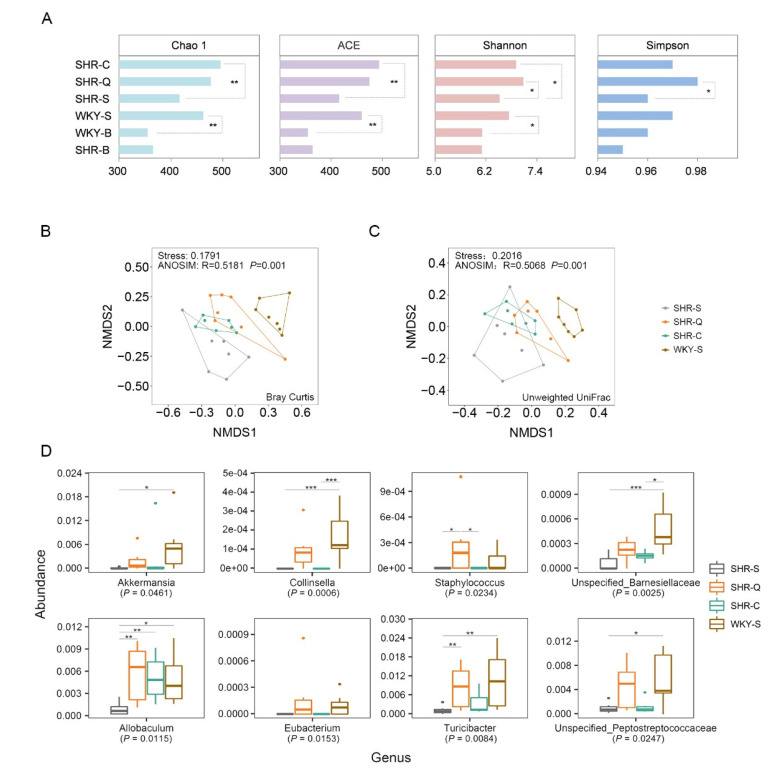
Significant differences in the fecal microbial structure of rats. SHR–S and WKY–S: rats administrated by saline solution for five weeks; SHR–Q and SHR–C: rats administrated by quinoa protein and captopril for five weeks, respectively. (**A**) Alpha diversity of fecal microbiota in different group rats. Significant differences are marked by * *p* < 0.05 and ** *p* < 0.01. Beta diversity of the fecal microbiota based on Bray–Curtis (**B**) and Unweighted UniFrac (**C**) distances by NMDS analysis. (**D**) Relative abundance of representative genera in fecal microbiota. *P* values denote the result of the Kruskal–Wallis comparison; markings above boxplots denote the Dunn’s post hoc result (with Bonferroni correction). Significant differences are marked by * P_adj_ < 0.1; ** P_adj_ < 0.05; *** P_adj_ < 0.01.

For microbial richness (Chao 1 and ACE) and diversity index (Shannon and Simpson), an increasing trend was observed in an age-dependent manner when SHR–B vs SHR–S and WKY–B vs WKY–S (Figure 3A). In addition, there was no significant difference (*p* > 0.05) in alpha diversity between SHRs and age-matched WKYs. Comparison of microbial communities was visualized by NMDS and the significant difference was evaluated by ANOSIM (Figure 2D). As presented in the plots, symbols representing the fecal samples were clustered according to their groups based on Bray–Curtis (NMDS Stress = 0.1383) and unweighted UniFrac distances (NMDS Stress = 0.1685), except for SHR–B and WKY–B partially overlapped. It is illustrated that the beta diversity of fecal microbiota differed in SHRs and WKYs of different ages, and this result was confirmed by the ANOSIM test with a *p*-value of 0.001.

Evidence for the gut microbiota to involve in the development of hypertension is plentifully available in the literature [33,34,35]. In this study, we confirmed that the microbial composition of SHRs differs from that of age-matched WKYs. At the age of 15 weeks, SHRs showed lower alpha diversity scores compared with WKYs, which is consistent with the previous report [35]. On the other hand, similar to the findings of Hoffman et al. [36], we reported a distinction between rats at different ages, i.e., the older rats (15-week-old) had higher alpha diversity than young rats (10-week-old). Aging has physiological effects on both the host and the microbiome, and age-related processes may influence the gut microbiota and its metabolites [37]. Therefore, age should be considered when comparing the results containing age-dependent changes. 

### 3.4. Improvement of Gut Microbial Dysbiosis in Treated SHRs 

The results presented in Figure 3A showed that SHR–Q and SHR–C had relatively higher alpha diversity in SHRs than SHR–S. In particular, SHR–Q showed significantly higher Shannon and Simpson indexes (*p* < 0.05), and SHR–C had a significant increase in Chao 1 and ACE compared with SHR–S (*p* < 0.01). Comparisons of alpha diversity between SHR–Q and SHR–C showed no significant differences (*p* > 0.05). The increase of gut microbial diversity is conducive to the development of a friendly microbial environment and the maintenance of normal physiological homeostasis [35].

According to beta diversity analysis based on Bray–Curtis (Figure 3B), the four groups formed a relatively separated distribution in the ordination space (NMDS Stress = 0.1789). Moreover, ANOSIM test based on Bray–Curtis (R = 0.5181, *p* = 0.001, Figure 3B) and unweighted UniFrac distances (R = 0.5068, *p* = 0.001, Figure 3C) also revealed significant differences in the overall microbial structure among different groups. Differences in the microbial structure were also calculated using pairwise PERMANOVA tests comparing each group to SHR–S (Table 2). The results showed that the community structure of the fecal microbiota in treated SHRs has undergone significant alternation measured by Bray–Curtis (*p* = 0.003 for both SHR–Q and SHR–C) and unweighted UniFrac distances (*p* = 0.012 for SHR–Q and *p* = 0.001 for SHR–C). 

**Figure 4:**
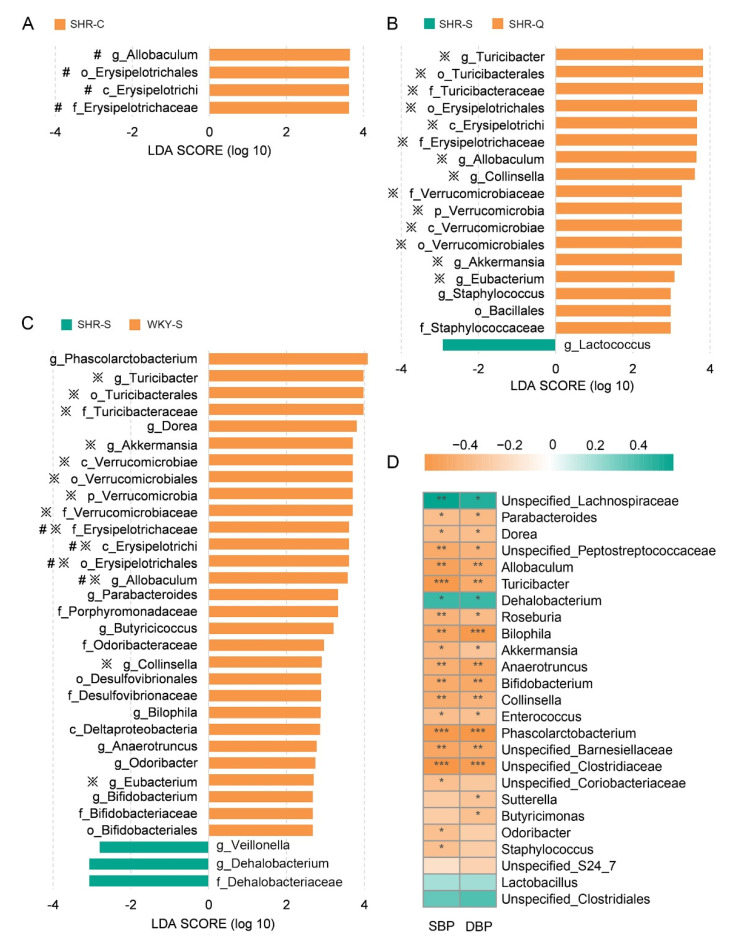
LEfSe analysis identifying taxonomic differences in fecal microbiotas. Histograms of LDA scores of microbiotas in (**A**) SHR–S vs SHR–C, (**B**) SHR–S vs SHR–Q, and (**C**) SHR–S vs WKY–S are shown with a cutoff value of LDA score above 2.0. Enriched taxa in SHR–S are indicated with a negative score (green), and taxa enriched in the SHR–C, SHR–Q, and WKY–S are characterized by a positive score (orange). The symbol “※” denotes the enriched taxon of both SHR–Q and WKY–S, and the symbol “#” denotes the enriched taxon of both SHR–C and WKY–S. (**D**) Heatmap of Spearman’s correlations between key microbiotas and blood pressure levels. Colors range from orange (negative correlation) to green (positive correlation). Significant correlations are marked by * *p* < 0.05, ** *p* < 0.01, *** *p*< 0.001.

To be specific, significant enrichment in the Verrucomicrobia phylum was observed in SHR–Q and SHR–C. At the genus level, the relative abundances of Akkermansia, Allobaculum, Collinsella, Eubacterium, Staphylococcus, Turicibacter, Unspecified_Barnesiellaceae, and Unspecified_Peptostreptococcaceae were significantly different among the groups (*p* < 0.05) (Figure 3D). Among these genera, Allobaculum (*p* < 0.05), Turicibacter (*p* < 0.05), and Staphylococcus (*p* < 0.1) in SHR–Q were observed significantly higher than that in SHR–S. Results from the LEfSe analysis further supported the statistical results. At the genus level, higher relative abundances were observed for g_Turicibacter, g_Allobaculum, g_Collinsella, g_Akkermansia, and g_Eubacterium both in SHR–Q (Figure 4B), and WKY–S (Figure 4C) when compared with SHR–S. Among them, g_Allobaculum was also enriched in SHR–C (Figure 4A). It is important to note that the fecal microbiota of SHR–Q shared more features in the composition of genera with that of WKY–S than SHR–C. 

### 3.5. Key Genera Related to Blood Pressure in Rats

Spearman’s rank correlation analysis was conducted to determine the association between gut microbiota and blood pressure levels. Twenty-two genera were found to closely correlate with blood pressure in rats (*p* < 0.05). As shown in Figure 4D, the SBP and DBP levels were positively correlated with the relative abundances of Dehalobacterium and Unspecified_Lachnospiraceae, but negatively with the other twenty genera. Consistent with this, Allobaculum, Turicibacter, Akkermansia, Collinsella, and Staphylococcus were found to be significantly enriched in SHR–Q comparing with SHR–S, as mentioned above. As for SHR–C, similar responses only occurred on Allobaculum. Previous research has reported that the Allobaculum genus is the most significant responder to captopril and is significantly associated with lowered blood pressure in SHRs [16], which is consistent with the results of our study. Akkermansia is known to exert a key role in host metabolic modulation, and its decline is proposed as a definitive biomarker of gut microbiota dysbiosis [38]. In this study, lower blood pressure level in SHR–Q was accompanied by a higher abundance of Akkermansia (belong to the phylum Verrucomicrobia) than the SHR–S. In agreement with our results, the study of Yang et al. [35] revealed that the minocycline treatment attenuates hypertension in chronic angiotensin-II induced hypertensive model, and also leads to the significant increase of phylum Verrucomicrobia and genus Akkermansia. Additionally, the abundance of Turicibacter had an obvious negative relationship with blood pressure levels in rats. Turicibacter has been reportedly depleted in rodent models with type 2 diabetes [39], tubulointerstitial fibrosis [40], and inflammatory bowel disease [41], which may have an important effect on host immune response [42]. Moreover, Turicibacter is correlated with the production of fecal short-chain fatty acids, and which present a crucial role in regulating blood pressure [43,44]. Together, treatment of hypertension led to increased abundance of several genera in the gut microenvironment of SHRs.

Based on the above results and explanation, it is suggested that quinoa protein is a good precursor of ACE inhibitory peptides and a promising candidate for the application in the treatment of hypertension. Then, the restoration and maintenance of normal blood pressure are conducive to the reconstruction of disordered microbial communities. It was worth noting that the microbial composition of SHR–Q shares more features with WKY–S than that of SHR–C, although there was no significant difference in blood pressure for SHR–Q and SHR–C. Therefore, this suggests that except for being affected by the blood pressure level, the alternation of gut microenvironment might also be affected by the antihypertension treatment alternative. Presumably, quinoa protein can interact directly with the gut microbiota. By changing the environmental conditions or providing nutrients for microbial growth [45], dietary protein may affect the gut microbial community structure and contribute to the construction of a friendly microbial environment, and then attenuate the development of hypertension. Although many published studies have examined the relationship between gut microbiota and hypertension, it is difficult to draw a definitive conclusion about the causal relationship between gut microbiota and hypertension [46]. It is quite possible that there is a bidirectional relationship between blood pressure and the gut microbiota, where the blood pressure level and the related pathological state contribute to the changes of the microbial community, and microbiota-derived metabolites contribute to the regulation of blood pressure. Gut microbial metabolites play a role in modulating host health [47]; microbial metabolites may contribute to the modulation of blood pressure by participating in the immune responses or inhibition of ACE activity [46]. Whatever alternation of microbiota dysbiosis is contributed by the improvement of blood pressure, or the interaction between microbiota and quinoa protein, it is important to note that antihypertension treatment by quinoa protein made the rats share, more common, with the non-hypertension one on microbial composition than drug treatment by captopril.

## 4. Conclusions

In summary, our observations demonstrated that quinoa protein is a promising natural source of ACE inhibitory peptides and is capable of lowering the blood pressure in SHRs, in which fecal microbiota has experienced a significant alteration, and towards that in non-hypertension rats. This study extends previous understanding of the antihypertension effect of quinoa protein and provides evidence that quinoa protein is linked to the regulation of hypertension-related gut dysbiosis. Further studies on the links of gut microbiota with blood pressure and quinoa protein will lead to a better understanding of the antihypertension role of quinoa protein. 

## Figures and Tables

**Table 1:** Main protein precursors of identified peptides, and potential ACE inhibitors in matched sequences using BIOPEP database.

Quinoa Protein	ACE Inhibitory Peptides
13S globulin seed storage protein 2-like: MSRVFLLPLALTLLILSPTSLAQLGFQLGQSPFLPSGQSSPQHSRLQRGQQALNDCQINQLSANEPSIRIQAEAGITEVWDPKEQQEFQCAGVTVIRREIEPKGLLLPHYNNAPSISYVIRGRGLLGLSSLGCADTYESGSPEFFSEESRRSERFEESRRSERGSEEMRDQHQKVRRFHKGHVIGLPAGVSKWVYNDGEDRLTIVTLYDTNNFQNQLDDNLRSFFLAGNPQGRGGDQSGRQHESSRRHTRGGQEEMGQNILSGFDKQLLADAFEVESDTISKIQGENDDRGAIIRVESGELEMLIPEWDQEEQRSERHHRGGGSERSEEEERSERHHRGGRGRQSESSRPHNGIEQTLCSARLSVNIDNPERADVFNPQGGRLTNINSNKLPILNYLRLSAEKVNLYKNAIMTPNWKINAHSIVYFTKGSGRVQIANHEGELVFDDMVQEGQLVVVPQNFVVLKRAGQDGLEWVALLTNDNAMSSPLAGRISAIRGMPIEVVMNSYKLSREEAQRLKYGRQELSVFSPSKRSERRGDEYAIV	LG, RL, LQ, GF, FQ, GQ, SG, PQ, LG, RL, LQIR, VW, KE, GL, LLP, PH, HY, YN, AP, NF, FQ, NLR, LR, SF, GG, GQ, MG, IL, SG, GF, FDK, EV, VE, QG, GE, IP, EW, EQR, ER, RA, NK, NKL, KLP, KL, IL, LN, LNY, NY, IA, EG, GE, LVF, VF, DM, PL, LA, AG, GR
legumin A-like: MAKSTTTLFLLSCSIALVLLNGCMGQGRMREMQGNECQIDRLTALEPTHRIQAEGGLTEVWDTQDQQFQCSGVSVIRRTIEPNGLLLPSFTSGPELIYIEQGNGISGLMIPGCPETFESMSQESWREGMERGMRGGRFQDQHQKIRHLRQGHIFAMPAGVAHWAYNSGNEPLVAVILIDTSNHANQLDKDYPKRFYLAGKPQQEHSRHHHRGGESQRGEHGSDGNVFSGLDTKSVAQSFGVSEDIAEKLQAKQDERGNIVLVQEGLHVIKPPSSRSYDDEREQRHRSPRSNGLEETICSARLSENIDDPSKADVYSPEAGRLTTLNSFNLPILSNLRLSAEKGVLYRNAIMAPHYNLNAHSIIYGVRGRGRIQIVNAQGNSVFDDELRQGQLVVVPQNFAVVKQAGEEGFEWIAFKTCENALFQTLAGRTSAIRAMPVEVISNIYQISREQAYRLKFSRSETTLFRPENQGRQRREMAA	ALEP, PT, AG, GV, AH, WA, AY, YN, SG, AG, GV, AH, WA, DY, YP, KR, RF, SF, SG, FG, GV, IA, IAE, EK, KL, LQ, GL, SF, SG, FG, GV, IA, IAE, EK, KL, LQ, GL, ER, RG, EG, GL, VIKP, IKP, KP, PP, KA, VYEA, AG, GR, RL, LN, SF, NLR, LR, RL, EK, KG, GV, GVLY, VLY, LY, AI, MAP, AP, PH, HY, YN, GR, VF, LR, GQ, QG, AV, EW, AVV, VK, AG, GE, EG, GF, EW, AI, IR, IRA, RA, LF, LFR, FR, RP, QG, GR
11S globulin seed storage protein 2-like: MGGTKILVALSLCLMVSSALGQGSHKRLSYQAQQCRINRLTSSEPNQRIECEGGLIELWDETEEQFQCSGIHAMRVTVQQNSLSLPNFHPFPRLVYIERGEGILGVTFPGCPETYDSSGRQEERIRGDEQREFGQQKDLHQKVHRFTRGDIIAIPPGAVHWCYNDGNEEVVTVIVNDLNNPSNQLDQTFRSFYLAGGLEKSSEIRGKINNIFRPFAPELLSEAFDVPEDLIRKMQQTENRGLIIRVDKGEMRILSPGSEQDYDDERRRKYVGLDVNGLEETICTMRLRHNLDNRREADVYSRHGGRLNIVNEHKLPILRHLDMSVEKGNMFPNTIYSPHWAVNSHSVVYVTRGEAHIQVVGNNGESVMDDRVNEGEMFVIPQYFTVSVKAGSNGFEYVSFKTTSSPMKSPMVGYTSVLRAMPVQVLTNAYQISPSEAHQLKYNREHQTFFLPSRGGRSRRF	NF, HP, PFP, FP, PR, RG, GD, IAIPP, IA, IAIP, AIPP, AI, AIP, IPP, IP, PP, PG, GA, AV, LN, TF, FR, EI, IR, RG, GK, IF, FR, RP, IR, TE, RG, GL, IL, LSP, PG, GS, DY, ER, HG, GG, GR, RL, LN, HK, KL, KLP, LPILR, IL, LR, VE, EK, KG, MF, FP, IY, IYSPH, PH, WA, AV, VG, GE, NG, VM, EG, VK, KA, AG, AGS, GS, NG, GF, EY, YV, SF, VG, GY, EA, AH, KY, YN

The protein fragments in red font were matched by identified peptides.

**Table 2:** Differences in beta-diversity of the fecal microbiota of rats between two groups were statistically assessed using PERMANOVA based on Bray–Curtis and unweighted UniFrac distances.

PERMANOVA (Pairwise Test)	*P*	
	Bray–Curtis	Unweighted UniFrac
SHR–S vs SHR–Q	0.003	0.012
SHR–S vs SHR–C	0.003	0.001
SHR–S vs WKY–S	0.001	0.001

## Data Availability

The data presented in this study are available on request from the corresponding author.

## Supplementary Materials

The following are available online at https://www.mdpi.com/article/10.3390/nu13072446/s1, Table S1: Identified peptides in digested samples.

## References

[1] Williams B., Mancia G., Spiering W., Agabiti Rosei E., Azizi M., Burnier M., Clement D.L., Coca A., de Simone G., Dominiczak A. (2018). ESC/ESH guidelines for the management of arterial hypertension. Eur. Heart J..

[2] Zhao D., Qi Y., Zheng Z., Wang Y., Zhang X.Y., Li H.J., Liu H.H., Zhang X.T., Du J., Liu J. (2011). Dietary factors associated with hypertension. Nat. Rev. Cardiol..

[3] Vasdev S., Stuckless J. (2010). Antihypertensive effects of dietary protein and its mechanism. Int. J. Angiol..

[4] Dasinger J.H., Fehrenbach D.J., Abais-Battad J.M. (2020). Dietary protein: Mechanisms influencing hypertension and renal disease. Curr. Hypertens. Rep..

[5] Ait-Yahia D., Madani S., Savelli J.L., Prost J., Bouchenak M., Belleville J. (2003). Dietary fish protein lowers blood pressure and alters tissue polyunsaturated fatty acid composition in spontaneously hypertensive rats. Nutrition.

[6] Pal S., Ellis V. (2010). The chronic effects of whey proteins on blood pressure, vascular function, and inflammatory markers in overweight individuals. Obesity.

[7] de Oliveira M.R., Silva T.J., Barros E., Guimarães V.M., Baracat-Pereira M.C., Eller M.R., Reis Coimbra J.S., de Oliveira E.B. (2018). Anti-Hypertensive Peptides Derived from Caseins: Mechanism of Physiological Action, Production Bioprocesses, and Challenges for Food Applications. Appl. Biochem. Biotechnol..

[8] Rai A.K., Sanjukta S., Jeyaram K. (2017). Production of angiotensin I converting enzyme inhibitory (ACE-I) peptides during milk fermentation and their role in reducing hypertension. Crit. Rev. Food Sci..

[9] Richter C.K., Skulas-Ray A.C., Champagne C.M., Kris-Etherton P.M. (2015). Plant protein and animal proteins: Do they differentially affect cardiovascular disease risk?. Adv. Nutr..

[10] Norris R., FitzGerald R.J., Hernandez-Ledesma B., Hsieh C.C. (2013). Antihypertensive peptides from food proteins. Bioactive Food Peptides in Health and Disease.

[11] Miralles B., Amigo L., Recio I. (2018). Critical review and perspectives on food derived antihypertensive peptides. J. Agric. Food Chem..

[12] Lee S.Y., Hur S.J. (2017). Antihypertensive peptides from animal products, marine organisms, and plants. Food Chem..

[13] Adnan S., Nelson J.W., Ajami N.J., Venna V.R., Petrosino J.F., Bryan R.M., Durgan D.J. (2017). Alterations in the gut microbiota can elicit hypertension in rats. Physiol. Genom..

[14] Marques F.Z., Mackay C.R., Kaye D.M. (2018). Beyond gut feelings: How the gut microbiota regulates blood pressure. Nat. Rev. Cardiol..

[15] Tain Y.L., Lee W.C., Wu K.L.H., Leu S., Chan J.Y.H. (2018). Resveratrol prevents the development of hypertension programmed by maternal plus post-weaning high-fructose consumption through modulation of oxidative stress, nutrient-sensing signals, and gut microbiota. Mol. Nutr. Food Res..

[16] Yang T., Aquino V., Lobaton G.O., Li H.B., Colon-Perez L., Goel R., Qi Y.F., Zubcevic J., Febo M., Richards E.M. (2019). Sustained captopril-induced reduction in blood pressure is associated with alterations in gut-brain axis in the spontaneously hypertensive rat. J. Am. Heart Assoc..

[17] Jama H.A., Beale A., Shihata W.A., Marques F.Z. (2019). The effect of diet on hypertensive pathology: Is there a link via gut microbiota-driven immunometabolism?. Cardiovasc. Res..

[18] Wu G.D., Chen J., Hoffmann C., Bittinger K., Chen Y.Y., Keilbaugh S.A., Bewtra M., Knights D., Walters W.A., Knight R. (2011). Linking long-term dietary patterns with gut microbial enterotypes. Science.

[19] Aluko R.E., Monu E. (2003). Functional and bioactive properties of quinoa seed protein hydrolysates. J. Food Sci..

[20] Mudgil P., Kilari B.P., Kamal H., Olalere O.A., FitzGerald R.J., Gan C.H., Maqsood S. (2020). Multifunctional bioactive peptides derived from quinoa protein hydrolysates: Inhibition of α-glucosidase, dipeptidyl peptidase-IV and angiotensin I converting enzymes. J. Cereal Sci..

[21] Ujiroghene O.J., Liu L., Zhang S.W., Lu J., Pang X.Y., Lv J.P. (2019). α-Glucosidase and ACE dual inhibitory protein hydrolysates and peptide fractions of sprouted quinoa yoghurt beverages inoculated with Lactobacillus casei. Food Chem..

[22] Zheng Y.J., Wang X., Zhuang Y.L., Li Y., Tian H.L., Shi P.Q., Li G.F. (2019). Isolation of novel ACE-inhibitory and antioxidant peptides from quinoa bran albumin assisted with an in silico approach: Characterization, in vivo antihypertension, and molecular docking. Molecules.

[23] Guo H.M., Hao Y.Q., Richel A., Everaert N., Chen Y.H., Liu M.J., Yang X.S., Ren G.X. (2020). Antihypertensive effect of quinoa protein under simulated gastrointestinal digestion and peptide characterization. J. Sci. Food Agric..

[24] Guo H.M., Richel A., Hao Y.Q., Everaert N., Yang X.S., Ren G.X. (2020). Novel dipeptidyl peptidase-IV and angiotensin-I-converting enzyme inhibitory peptides released from quinoa protein by in silico proteolysis. Food Sci. Nutr..

[25] Fritz M., Rinaldi G. (2008). Blood pressure measurement with the tail-cuff method in Wistar and spontaneously hypertensive rats: Influence of adrenergic- and nitric oxide-mediated vasomotion. J. Pharmacol. Toxicol. Methods.

[26] Boutrou R., Gaudichon C., Dupont D., Jardin J., Airinei G., Marsset-Baglieri A., Benamouzig R., Tome D., Leonil J. (2013). Sequential release of milk protein-derived bioactive peptides in the jejunum in healthy humans. Am. J. Clin. Nutr..

[27] Boutrou R., Henry G., Sanchez-Rivera L. (2015). On the trail of milk bioactive peptides in human and animal intestinal tracts during digestion: A review. Dairy Sci. Technol..

[28] Portune K.J., Beaumont M., Davila A.M., Tomé D., Blachier F., Sanz Y. (2016). Gut microbiota role in dietary protein metabolism and health-related outcomes: The two sides of the coin. Trends Food Sci. Technol..

[29] Davila A.M., Blachier F., Gotteland M., Andriamihaja M., Benetti P.H., Sanz Y., Tomé D. (2013). Intestinal luminal nitrogen metabolism: Role of the gut microbiota and consequences for the host. Pharmacol. Res..

[30] Barbe F., Le Feunteun S., Remond D., Ménard O., Jardin J., Henry G., Laroche B., Dupont D. (2014). Tracking the in vivo release of bioactive peptides in the gut during digestion: Mass spectrometry peptidomic characterization of effluents collected in the gut of dairy matrix. Food Res. Int..

[31] Volpe M., Gallo G., Tocci G. (2018). Is early and fast blood pressure control important in hypertension management?. Int. J. Cardiol..

[32] Gupta R., Guptha S. (2010). Strategies for initial management of hypertension. Indian J. Med. Res..

[33] Mell B., Jala V.R., Mathew A.V., Byun J., Waghulde H., Zhang Y., Haribabu B., Vijay-Kumar M., Pennathur S., Joe B. (2015). Evidence for a link between gut microbiota and hypertension in the Dahl rat. Physiol. Genom..

[34] Durgan D.J., Ganesh B.P., Cope J.L., Ajami N.J., Phillips S.C., Petrosino J.F., Hollister E.B., Bryan R.M. (2016). Role of the gut microbiome in obstructive sleep apnea-induced hypertension. Hypertension.

[35] Yang T., Santisteban M.M., Rodriguez V., Li E., Ahmari N., Marulanda Carvajal J., Zadeh M., Gong M., Qi Y., Zubcevic J. (2015). Gut dysbiosis is linked to hypertension. Hypertension.

[36] Hoffman J.D., Parikh I., Green S.J., Chlipala G., Mohney R.P., Keaton M., Bauer B., Hartz A.M.S., Lin A.L. (2017). Age drives distortion of brain metabolic, vascular and cognitive functions, and the gut microbiome. Front. Aging Neurosci..

[37] Badal V.D., Vaccariello E.D., Murray E.R., Yu K.E., Knight R., Jeste D.V., Nguyen T.T. (2020). The gut microbiome, aging, and longevity: A systematic review. Nutrients.

[38] Lopetuso L.R., Quagliariello A., Schiavoni M., Petito V., Russo A., Reddel S., Chierico F.D., Ianiro G., Scaldaferri F., Neri M. (2020). Towards a disease-associated common trait of gut microbiota dysbiosis: The pivotal role of Akkermansia muciniphila. Digest. Liver Dis..

[39] Liu G., Bei J., Liang L., Yu G., Li L., Li Q. (2018). Stachyose improves inflammation through modulating gut microbiota of high-fat diet/streptozotocin-induced type 2 diabetes in rats. Mol. Nutr. Food Res..

[40] Chen L., Chen D.Q., Liu J.R., Zhang J., Vaziri N.D., Zhuang S., Chen H., Feng Y.L., Guo Y., Zhao Y.Y. (2019). Unilateral ureteral obstruction causes gut microbial dysbiosis and metabolome disorders contributing to tubulointerstitial fibrosis. Exp. Mol. Med..

[41] Gu Z., Zhu Y., Jiang S., Xia G., Li C., Zhang X., Zhang J., Shen X. (2020). Tilapia head glycolipids reduce inflammation by regulating the gut microbiota in dextran sulphate sodium-induced colitis mice. Food Funct..

[42] Presley L.L., Wei B., Braun J., Borneman J. (2010). Bacteria associated with immunoregulatory cells in mice. Appl. Environ. Microbiol..

[43] Zhong Y., Nyman M., Fak F. (2015). Modulation of gut microbiota in rats fed high-fat diets by processing whole-grain barley to barley malt. Mol. Nutr. Food Res..

[44] Naqvi S., Asar T.O., Kumar V., Al-Abbasi F.A., Alhayyani S., Kamal M.A., Anwar F. (2021). A cross-talk between gut microbiome, salt and hypertension. Biomed. Pharmacother..

[45] Metzler-Zebeli B.U., Mann E., Schmitz-Esser S., Wagner M., Ritzmann M., Zebelib Q. (2013). Changing dietary calcium-phosphorus level and cereal source selectively alters abundance of bacteria and metabolites in the upper gastrointestinal tracts of weaned pigs. Appl. Environ. Microbiol..

[46] Al Khodor S., Reichert B., Shatat I.F. (2017). The microbiome and blood pressure: Can microbes regulate our blood pressure?. Front. Pediatr..

[47] Zhang L.S., Davies S.S. (2016). Microbial metabolism of dietary components to bioactive metabolites: Opportunities for new therapeutic interventions. Genome Med..

