# Supplementary material for: Administration with Quinoa Protein Reduces the Blood Pressure in Spontaneously Hypertensive Rats and Modifies the Fecal Microbiota
Source: Nutrients. 2021 Jul 17;13(7):2446. doi: 10.3390/nu13072446 (PMC8308759; doi:10.3390/nu13072446)
Supplement: Supplementary file 1 [file nutrients-13-02446-s001.zip › nutrients-1267633-supplementary.pdf]

Table S1 Identified peptides in digested samples

Stomach contents at 0.5 h				
**No.**	**Sequence**	**Spectra**	**Protein (Unused Score ⩾2.0)**	**Protein Accession**
1	FTVSVKAGSNGFE	8	11S globulin seed storage protein 2-like	XP_021768838.1
2	ILSPGSEQDYDDER	6	11S globulin seed storage protein 2-like	XP_021768838.1
3	IQVVGNNGESVMDDRVNEG	3	11S globulin seed storage protein 2-like	XP_021768838.1
4	IQVVGNNGESVMDDRVNEGEM	4	11S globulin seed storage protein 2-like	XP_021768838.1
5	IRKMQQTENRGL	1	11S globulin seed storage protein 2-like	XP_021768838.1
6	PNTIYSPHWAVNSH	1	11S globulin seed storage protein 2-like	XP_021768838.1
7	PNTIYSPHWAVNSHSV	1	11S globulin seed storage protein 2-like	XP_021768838.1
8	PNTIYSPHWAVNSHSVV	2	11S globulin seed storage protein 2-like	XP_021768838.1
9	SSEIRGKINNIFR	4	11S globulin seed storage protein 2-like	XP_021768838.1
10	SSEIRGKINNIFRP	1	11S globulin seed storage protein 2-like	XP_021768838.1
11	SVEKGNMFPNTIYSPHWAVNSH	1	11S globulin seed storage protein 2-like	XP_021768838.1
12	SVEKGNMFPNTIYSPHWAVNSHS	1	11S globulin seed storage protein 2-like	XP_021768838.1
13	TRGDIIAIPPGAVH	13	11S globulin seed storage protein 2-like	XP_021768838.1
14	VIVNDLNNPSNQLDQTFR	3	11S globulin seed storage protein 2-like	XP_021768838.1
15	VIVNDLNNPSNQLDQTFRS	1	11S globulin seed storage protein 2-like	XP_021768838.1
16	VQQNSLSLPNFH	5	11S globulin seed storage protein 2-like	XP_021768838.1
17	YQISPSEAHQLKYNR	1	11S globulin seed storage protein 2-like	XP_021768838.1
18	HGGRLNIVNE	1	11S globulin seed storage protein 2-like	XP_021768838.1
19	INNIFRPFA	1	11S globulin seed storage protein 2-like	XP_021768838.1
20	SVEKGNMFPNTIYSPHW	1	11S globulin seed storage protein 2-like	XP_021768838.1
21	RILSPGSEQD	1	11S globulin seed storage protein 2-like	XP_021768838.1
22	PNTIYSPHW	2	11S globulin seed storage protein 2-like	XP_021768838.1
23	QNSLSLPNFHP	1	11S globulin seed storage protein 2-like	XP_021768838.1
24	HGGRLNIVNEHK	1	11S globulin seed storage protein 2-like	XP_021768838.1
25	SLSLPNFHPFPR	1	11S globulin seed storage protein 2-like	XP_021768838.1
26	VQQNSLSLPNFHP	1	11S globulin seed storage protein 2-like	XP_021768838.1
27	IRGKINNIFR	1	11S globulin seed storage protein 2-like	XP_021768838.1
28	GGRLNIVNEHKLPILRH	1	11S globulin seed storage protein 2-like	XP_021768838.1
29	DTNNFQNQLDDNLR	5	13S globulin seed storage protein 2-like	XP_021752668.1
30	FQLGQSPFLPSGQSSPQH	1	13S globulin seed storage protein 2-like	XP_021752668.1
31	FQLGQSPFLPSGQSSPQHS	1	13S globulin seed storage protein 2-like	XP_021752668.1
32	GGQEEMGQNILSGFDK	1	13S globulin seed storage protein 2-like	XP_021752668.1
33	GLLLPHYNNAPSIS	1	13S globulin seed storage protein 2-like	XP_021752668.1
34	IANHEGELVFDDM	1	13S globulin seed storage protein 2-like	XP_021752668.1
35	LLTNDNAMSSPLAGR	12	13S globulin seed storage protein 2-like	XP_021752668.1
36	LLTNDNAMSSPLAGRIS	14	13S globulin seed storage protein 2-like	XP_021752668.1
37	LSVNIDNPERAD	1	13S globulin seed storage protein 2-like	XP_021752668.1
38	LTNDNAMSSPLAGR	6	13S globulin seed storage protein 2-like	XP_021752668.1
39	LTNDNAMSSPLAGRIS	14	13S globulin seed storage protein 2-like	XP_021752668.1
40	NAMSSPLAGRIS	5	13S globulin seed storage protein 2-like	XP_021752668.1
41	QSPFLPSGQSSPQHSR	1	13S globulin seed storage protein 2-like	XP_021752668.1
42	QSPFLPSGQSSPQHSRLQ	1	13S globulin seed storage protein 2-like	XP_021752668.1
43	VALLTNDNAMSSPLAGR	4	13S globulin seed storage protein 2-like	XP_021752668.1
44	VALLTNDNAMSSPLAGRIS	1	13S globulin seed storage protein 2-like	XP_021752668.1
45	YDTNNFQNQLDDNLR	14	13S globulin seed storage protein 2-like	XP_021752668.1
46	YDTNNFQNQLDDNLRS	8	13S globulin seed storage protein 2-like	XP_021752668.1
47	MLIPEWDQEEQR	1	13S globulin seed storage protein 2-like	XP_021752668.1
48	QLGFQLGQSPFLPSGQSSPQHSR	1	13S globulin seed storage protein 2-like	XP_021752668.1
49	LSVNIDNPER	2	13S globulin seed storage protein 2-like	XP_021752668.1
50	SANEPSIRIQ	3	13S globulin seed storage protein 2-like	XP_021752668.1
51	GGQEEMGQNILSGFDKQ	1	13S globulin seed storage protein 2-like	XP_021752668.1
52	GQSPFLPSGQSSPQHSRLQ	2	13S globulin seed storage protein 2-like	XP_021752668.1
53	GLLLPHYNNAP	1	13S globulin seed storage protein 2-like	XP_021752668.1
54	LTNINSNKLPILN	1	13S globulin seed storage protein 2-like	XP_021752668.1
55	NDNAMSSPLAGRIS	6	13S globulin seed storage protein 2-like	XP_021752668.1
56	EVESDTISKIQGE	4	13S globulin seed storage protein 2-like	XP_021752668.1
57	DDELRQGQL	1	legumin A-like	XP_021770181.1

58	GRIQIVNAQGNSVFDDELR	1	legumin A-like	XP_021770181.1
59	GRIQIVNAQGNSVFDDELRQG	1	legumin A-like	XP_021770181.1
60	GVSEDIAEKLQAK	3	legumin A-like	XP_021770181.1
61	GVSEDIAEKLQAKQDERGNIV	4	legumin A-like	XP_021770181.1
62	IQIVNAQGNSVFDDELRQG	1	legumin A-like	XP_021770181.1
63	IVNAQGNSVFDDELR	5	legumin A-like	XP_021770181.1
64	IVNAQGNSVFDDELRQG	8	legumin A-like	XP_021770181.1
65	NIDDPSKADVYSPEAGRLT	2	legumin A-like	XP_021770181.1
66	RNAIMAPHYN	3	legumin A-like	XP_021770181.1
67	SAEKGVLYRNA	1	legumin A-like	XP_021770181.1
68	SGLDTKSVAQS	1	legumin A-like	XP_021770181.1
69	YRNAIMAPHYN	16	legumin A-like	XP_021770181.1
70	GRIQIVNAQGNSVFDDELRQGQL	1	legumin A-like	XP_021770181.1
71	LFRPENQGR	1	legumin A-like	XP_021770181.1
72	VQEGLHVIKPPSSR	2	legumin A-like	XP_021770181.1
73	RTSAIRAMP	1	legumin A-like	XP_021770181.1
74	AMPAGVAHWAYNSGNEP	1	legumin A-like	XP_021770181.1
75	HANQLDKDYPKRF	1	legumin A-like	XP_021770181.1
76	QAEQDERGNIV	1	legumin A-like	XP_021768828.1
77	TALEPTNRIQ	1	legumin A-like	XP_021768828.1
78	AMPAGVAHWAYNTGNEP	1	legumin A-like	XP_021768828.1
79	NIDEPSKADVYSPEAGRLT	1	legumin A-like	XP_021768828.1
80	GVSEDIAEKLQAEQDERGNIV	1	legumin A-like	XP_021768828.1
81	EEEESSGKGRPYV	2	vicilin-like antimicrobial peptides 2-2	XP_021764389.1
82	EEESSGKGRPYV	1	vicilin-like antimicrobial peptides 2-2	XP_021764389.1
83	INPVSNIGKFE	5	vicilin-like antimicrobial peptides 2-2	XP_021764389.1
84	SQQREGAIIKASEEQIR	4	vicilin-like antimicrobial peptides 2-2	XP_021764389.1
85	VAFANITQGSMHTPYYN	1	vicilin-like antimicrobial peptides 2-2	XP_021764389.1
86	VAFANITQGSMHTPYYNSR	1	vicilin-like antimicrobial peptides 2-2	XP_021764389.1
87	VKLINPVSNIGK	1	vicilin-like antimicrobial peptides 2-2	XP_021764389.1
88	FANITQGSMHTPYYNSR	1	vicilin-like antimicrobial peptides 2-2	XP_021764389.1
89	FKTSEDSLK	1	vicilin-like antimicrobial peptides 2-2	XP_021764389.1
90	AENNHKFPLAGQR	2	vicilin-like antimicrobial peptides 2-2	XP_021764389.1
91	LINPVSNIGKFE	1	vicilin-like antimicrobial peptides 2-2	XP_021764389.1
92	SQQREGAIIKASE	1	vicilin-like antimicrobial peptides 2-2	XP_021764389.1
93	VAFANITQGSMHTPY	1	vicilin-like antimicrobial peptides 2-2	XP_021764389.1
94	VAFANITQGSMHTP	1	vicilin-like antimicrobial peptides 2-2	XP_021764389.1
95	VSFANITQGSMHTPY	1	vicilin-like antimicrobial peptides 2-1	XP_021762713.1
96	VSFANITQGSMHTPYYNS	1	vicilin-like antimicrobial peptides 2-1	XP_021762713.1
97	VSFANITQGSMHTPYYNSR	3	vicilin-like antimicrobial peptides 2-1	XP_021762713.1
98	VSTPGVLPVASGGIHV	1	phosphate carboxylase/oxygenase large subu	YP_009380136.1
99	VSTPGVLPVASGGIHVW	1	phosphate carboxylase/oxygenase large subu	YP_009380136.1
100	RVQEGDVIGSPAGVVQWT	1	13S globulin seed storage protein 1-like	XP_021752233.1
101	DESGPSIVHRKC	1	actin-1	XP_021749538.1
102	SYELPDGQVITIGAER	1	actin-1	XP_021749538.1
103	HTGPGILSMANAGPNTNGSQF	2	peptidyl-prolyl cis-trans isomerase 1	XP_021775867.1
104	NVINGGSHAGNKLA	1	enolase	XP_021743148.1
105	VDLEPTVIDEVRTGT	1	tubulin alpha-3 chain	XP_021714947.1
106	AGQKTKDVGQTIESK	1	oleosin 18.2 kDa-like	XP_021732650.1
107	SVIGAIGSTYEHAK	2	bryogenesis abundant protein ECP63-like iso	XP_021737795.1
108	ERLLGAALGSALTAAVV	9	uncharacterized protein LOC110711964	XP_021746098.1

Stomach contents at 1.0 h

No.	Sequence	Spectra	Quinoa Protein (Unused Score ⩾2.0)	Protein Accession
1	AVVKQAGEEGFE	1	legumin A-like	XP_021770181.1
2	IDTSNHANQLDK	1	legumin A-like	XP_021770181.1
3	IDTSNHANQLDKDYPKRF	1	legumin A-like	XP_021770181.1
4	IVNAQGNSVFDDELRQG	1	legumin A-like	XP_021770181.1
5	IVNAQGNSVFDDELRQGQL	1	legumin A-like	XP_021770181.1
6	NIDDPSKADVYSPEAGRLT	2	legumin A-like	XP_021770181.1
7	NIDDPSKADVYSPEAGRLTTL	1	legumin A-like	XP_021770181.1
8	SAEKGVLYR	1	legumin A-like	XP_021770181.1
9	SAEKGVLYRN	1	legumin A-like	XP_021770181.1

10	SAEKGVLYRNAIM	2	legumin A-like	XP_021770181.1
11	SGLDTKSVAQS	1	legumin A-like	XP_021770181.1
12	SGLDTKSVAQSF	3	legumin A-like	XP_021770181.1
13	SPEAGRLTTLNS	1	legumin A-like	XP_021770181.1
14	SPEAGRLTTLNSFN	1	legumin A-like	XP_021770181.1
15	TALEPTHRIQ	1	legumin A-like	XP_021770181.1
16	SNLRLSAEKG	1	legumin A-like	XP_021770181.1
17	YRNAIMAPHYN	1	legumin A-like	XP_021770181.1
18	HANQLDKDYPKRF	1	legumin A-like	XP_021770181.1
19	FTVSVKAGSNGFE	1	11S globulin seed storage protein 2-like	XP_021768838.1
20	FTVSVKAGSNGFEY	2	11S globulin seed storage protein 2-like	XP_021768838.1
21	ILSPGSEQDYDDER	1	11S globulin seed storage protein 2-like	XP_021768838.1
22	IQVVGNNGESVMDDRVNEG	2	11S globulin seed storage protein 2-like	XP_021768838.1
23	PNTIYSPHW	1	11S globulin seed storage protein 2-like	XP_021768838.1
24	VSFKTTSSPMKSPMVGY	1	11S globulin seed storage protein 2-like	XP_021768838.1
25	VTVQQNSLSLPNFHP	1	11S globulin seed storage protein 2-like	XP_021768838.1
26	VVGNNGESVMDDRVNEG	1	11S globulin seed storage protein 2-like	XP_021768838.1
27	VVGNNGESVMDDRVNEGEM	1	11S globulin seed storage protein 2-like	XP_021768838.1
28	VTVQQNSLSLPNFHPF	1	11S globulin seed storage protein 2-like	XP_021768838.1
29	LLTNDNAMSSPLAGRIS	4	13S globulin seed storage protein 2-like	XP_021752668.1
30	LTNDNAMSSPLAGRIS	2	13S globulin seed storage protein 2-like	XP_021752668.1
31	VALLTNDNAMSSPLAGRIS	1	13S globulin seed storage protein 2-like	XP_021752668.1
32	VWDPKEQQE	1	13S globulin seed storage protein 2-like	XP_021752668.1
33	YDTNNFQNQLDDNLR	3	13S globulin seed storage protein 2-like	XP_021752668.1
34	EEEESSGKGRPY	1	vicilin-like antimicrobial peptides 2-1	XP_021762713.1
35	LINPVSNIGKFE	1	vicilin-like antimicrobial peptides 2-1	XP_021762713.1
36	NIERGHVM	1	vicilin-like antimicrobial peptides 2-1	XP_021762713.1
37	EEEESSGKGRPYV	1	vicilin-like antimicrobial peptides 2-1	XP_021762713.1
38	LTYYTPEYETQ	3	phosphate carboxylase/oxygenase large subu	YP_009380136.1
39	AGVKDYK	1	phosphate carboxylase/oxygenase large subu	YP_009380136.1
40	TFQGPPHGIQVERD	1	phosphate carboxylase/oxygenase large subu	YP_009380136.1
41	LRVAPEEHPVL	1	actin-66-like	XP_021716156.1
42	NIIPSSTGAAKAVGKVL	1	PROTEIN: glyceraldehyde-3-phosphate deh	XP_021736301.1
43	ELIDSPNNKGWKP	1	ankyrin-3-like	XP_021744366.1
44	IIANDQGNRTTPSY	2	heat shock cognate 70 kDa protein 2	XP_021714917.1
45	VIIMNHPGQIGNGY	1	elongation factor 1-alpha	XP_021744217.1
46	GADAAGLLLRE	1	r of RNA polymerase II transcription subuni	XP_021726416.1
47	KYDEIDAAPEER	1	elongation factor TuB, chloroplastic-like	XP_021733533.1
48	IQQATGGTHATQQSS	1	oleosin 1-like	XP_021721762.1
49	ETTENESANEGYR	1	PsbA (chloroplast)	YP_009380110.1
50	RVQEGDVIGSPAGVAQ	1	13S globulin seed storage protein 1-like	XP_021721911.1
51	LGANVGSAQGPTGLGK	1	PsbC (chloroplast)	YP_009380126.1
52	TAENFSNATGEQGYPGGK	1	rophyll a-b binding protein CP24, chloropla	XP_021751200.1
53	FVQQATGGTHATQQSS	1	oleosin 1-like	XP_021752156.1

Stomach contents at 2.0 h

No.	Sequence	Spectra	Quinoa Protein (Unused Score ≥2.0)	Protein Accession
1	LRVAPEEHPVL	3	actin-11	XP_021744491.1
2	VGDEAQSKRGIL	1	actin-11	XP_021744491.1
3	VGDEAQSKRGILTLKY	1	actin-11	XP_021744491.1
4	RLDLAGRDLTDY	1	actin-11	XP_021744491.1
5	LTYYTPEYETQ	2	phosphate carboxylase/oxygenase large subu	YP_009380136.1
6	AGVKDYK	5	phosphate carboxylase/oxygenase large subu	YP_009380136.1
7	TFQGPPHGIQVERD	1	phosphate carboxylase/oxygenase large subu	YP_009380136.1
8	IIANDQGNRTTPSY	3	heat shock cognate 70 kDa protein 2	XP_021714917.1
9	TKMKEIAE	1	heat shock cognate 70 kDa protein 2	XP_021714917.1
10	TGVLKPGMVVTF	1	elongation factor 1-alpha-like	XP_021715816.1
11	ASKSNGKRSNVEAI	3	guanylate-binding protein 2-like	XP_021722291.1
12	HVLTSSILNAVRK	1	putative zinc transporter At3g08650	XP_021723322.1
13	RIGTGLFIATF	1	protein NRT1/ PTR FAMILY 4.3-like	XP_021713499.1
14	SPGYYDGR	1	sphosphate carboxylase small chain 1, chloro	XP_021755123.1
15	LENVIRDAVTY	2	histone H4	XP_021713480.1
16	NVINGGSHAGNKLAMQEF	1	enolase	XP_021743148.1

17	ETTENESANEGYR	3	PsbA (chloroplast)	YP_009380110.1
**Small intestine contents at 0.5 h**				
**No.**	**Sequence**	**Spectra**	**Quinoa Protein (Unused Score ⩾2.0)**	**Protein Accession**
1	LTNINSNKLPILNYL	1	13S globulin seed storage protein 2-like	XP_021752668.1
2	SETTLFRPENQG	1	legumin A-like	XP_021768828.1
3	SKQKISSLKPVE	1	haracterized protein LOC110736894 isoform	XP_021772925.1
4	GKSLEQVLEIT	1	hexokinase-2-like	XP_021749832.1
**Small intestine contents at 1.0 h**				
**No.**	**Sequence**	**Spectra**	**Quinoa Protein (Unused Score ⩾2.0)**	**Protein Accession**
1	YDTNNFQNQLDDNLRSF	1	13S globulin seed storage protein 2-like	XP_021752668.1
1	DTNNFQNQLDDNLRSF	1	13S globulin seed storage protein 2-like	XP_021752668.1
2	AVVKQAGEEGFEW	1	legumin A-like	XP_021768828.1

* No peptide was identified in small intestine contents at 2.0 h and the colon contents.
